# Investigation of the Relationship Between Hip and Knee Osteoarthritis and Disordered Spinal and Pelvic Morphology

**DOI:** 10.7759/cureus.20861

**Published:** 2022-01-01

**Authors:** Vasileios A Kechagias, Theodoros B Grivas, Panayiotis J Papagelopoulos, Vasileios A Kontogeorgakos, Konstantinos Vlasis

**Affiliations:** 1 Orthopaedics, General Hospital of Lemnos, Lemnos, GRC; 2 Orthopaedics and Traumatology, Tzaneio General Hospital of Piraeus, Piraeus, GRC; 3 First Department of Orthopaedics, Attikon University General Hospital, National and Kapodistrian University of Athens School of Medicine, Athens, GRC; 4 Department of Anatomy, National and Kapodistrian University of Athens School of Medicine, Athens, GRC

**Keywords:** osteoarthritis and disordered trunk, total knee arthroplasty, total hip arthroplasty, knee osteoarthritis, hip osteoarthritis

## Abstract

Introduction

A critical question is the causal relationship between hip or knee osteoarthritis (OA) and disordered spinal and pelvic morphology. The aim of this study is to examine this correlation. Therefore, we studied the effect of total hip or knee arthroplasty (THA/TKA) on truncal parameters to determine the causal relationship between these two situations.

Materials and methods

This is a prospective study of the effect of THA or TKA in patients with hip or knee OA on truncal morphological parameters. Patients with one-sided hip or knee OA who chose to undergo THA or TKA were enrolled and surveyed. A control group (CG) was also surveyed for comparison with the patients. The patients were preoperatively examined for truncal parameters using the Diers Formetric four-D analysis system (surface topography technique) to calculate several truncal parameters in all planes at four months and 12 months postoperatively. Measurable examinations were performed using the Statistical Package for the Social Sciences (SPSS) version 17.00 (SPSS Inc., Chicago), and statistical significance was set at a p-value of <0.05.

Results

The study examined 34 patients who underwent THA, including 19 women and 15 men with a mean age of 67.62 ± 8.28 years. The study also examined 45 patients who underwent TKA, including 34 women and 11 men, with a mean age of 72.42 ± 7.0 years. These patients were also compared with a CG that consisted of 25 normal individuals, including 12 women and 13 men, with a mean age of 69.28 ± 10.11 years.

The results of this study from four months after THA revealed that the lordotic angle, trunk torsion, pelvic inclination, pelvic obliquity, and pelvis rotation were improved to normal levels. At 12 months after THA, only the pelvic obliquity was improved to normal levels. At four months after TKA the lordotic angle, pelvic inclination, and pelvic obliquity were improved to normal levels. However, the fleche cervicale and vertebral rotation were worse. At 12 months after TKA, only the pelvic obliquity was improved to normal levels.

Conclusions

THA and TKA to correct hip and knee OA do not correct the disordered morphology of the trunk in the long term. Thus, hip or knee OA does not seem to be responsible for disordered trunk morphology. However, it cannot be ruled out whether the disturbed morphology is responsible for the appearance of the hip and knee OA.

## Introduction

It is known from many studies that patients with hip and knee osteoarthritis (OA) have disordered spinal and pelvic morphology [1-7]. A critical question of concern in the orthopedic community is what the relationship is between hip and knee OA and disordered spinal and pelvic morphology. Specifically, it is unknown whether there is a causal relationship between them, which of the two situations precedes the other, and whether one causes the other. These questions have not been answered in recent studies [5,8,9].

The present study attempts to answer these questions by examining the impact of total hip or knee arthroplasty (THA/TKA) in patients with hip or knee OA on truncal morphological parameters. The same groups of patients with hip or knee OA have already been studied and compared with a control group (CG), and disturbed trunk morphologies have been found in both groups of patients preoperatively [1].

Thus, we assumed that if we have improvement of the truncal parameters after THA or TKA in the long term to correct hip or knee OA, then the hip and knee OA could be the cause of changes in the morphology of the spine and pelvis. In the opposite case, if the changes of the truncal parameters are still present after THA and TKA, then this could exclude that OA is responsible for the appearance of the disordered morphology of the trunk. However, it could not be determined whether the disturbed morphology of the trunk is responsible for OA.

## Materials and methods

Study design

This is a prospective study of the effect of THA or TKA in patients with hip or knee OA on truncal morphological parameters. Patients with one-sided hip or knee OA who chose to undergo THA or TKA at Tzaneio General Hospital of Piraeus were enrolled and surveyed. A CG was also surveyed for comparison with the patients. The study was approved by Attikon University General Hospital's Institutional Review Board (ΕΒΔ390/ 9-9-2014, date of approval 24-9-2014). The principles of the Declaration of Helsinki were applied throughout the study. Informed consent was obtained from all participants.

Inclusion and exclusion criteria

The following inclusion criteria of the CG were applied: (1) without OA in the joints of lower extremities, (2) without neurological deficits in lower extremities, (3) without a history of surgical intervention in the spine or lower extremities, and (4) without other diseases that would affect the alignment of the trunk. The following exclusion criteria of the patients were applied: (1) marked OA in other joints of lower extremities, (2) arthritis secondary to other diseases, e.g., ankylosing spondylitis, rheumatoid arthritis, developmental dysplasia, and trauma, (3) neurological deficits in lower extremities, (4) history of surgical intervention in the spine or lower extremities, and (5) other diseases that would affect the alignment of the trunk.

Data collection

The patients were preoperatively examined for truncal parameters using the Diers Formetric four-D analysis system (surface topography technique) to calculate several truncal parameters in all planes and at four months and 12 months postoperatively. All the calculations were performed using the Statistical Package for the Social Sciences (SPSS) version 17.00 (SPSS Inc., Chicago), and statistical significance was set at a p-value of <0.05.

## Results

Control group

The CG comprised of 25 normal individuals with 12 women, 13 men, with a mean age of 69.28 ± 10.11 years (range, 55-86 years). They had a mean weight of 79.40 ± 13.08 kg, a mean height of 165.04 ± 9.46 mm, and a mean body mass index (BMI) of 29.00 ± 3.00 kg/m^2^. The normal values of the CG are for the fleche cervicale (mm; 79.02), fleche lombaire (mm; 39.41), kyphotic angle (°; 56.18), lordotic angle (°; 42.26), sagittal imbalance (°; 4.19), sagittal imbalance (mm; 32.88), coronal imbalance (°; 1.16), coronal imbalance (mm; 8.90), apical deviation root mean square (rms) (mm; 4.94), apical deviation amplitude (mm; 11.40), apical deviation max (mm; 9.20), scoliosis angle (°; 12.96), vertebral rotation rms (°; 3.98), vertebral rotation amplitude (°; 9.64), vertebral rotation max (°; 7.80), trunk torsion (°; 4.16), pelvic inclination symmetry line (°; 19.04), pelvic inclination dimples (°; 17.44), pelvic torsion (°; 2.64), pelvic obliquity (°; 0.96), pelvic obliquity (mm; 1.34), and pelvis rotation (°; 2.08). 

Patients with THA

A group of 34 patients with 19 women, 15 men, and a mean age of 67.62 ± 8.28 years (range, 47-84 years) was surveyed preoperatively. Of these patients, 15 were surveyed at four and 12 months postoperatively, 14 patients were surveyed only at four months postoperatively, and five patients were surveyed only at 12 months postoperatively. There were 20 patients who underwent an operation for the right hip, and 14 underwent one for the left hip. Four orthopedic surgeons carried out THA. Other characteristics of these patients include a mean weight of 82.32 ± 17.73 kg, a mean height of 165.79 ± 8.80 mm, and a mean BMI of 29.72 ± 4.31 kg/m^2^. Table 1 summarizes the homogeneity of the demographic characteristics between the CG and the patients with THA. No statistically significant differences were noted between the CG and patients with THA.

**Table 1:** Homogeneity of demographic characteristics between the CG and patients with THA CG: control group, THA: total hip arthroplasty, SD: standard deviation, BMI: body mass index

	Characteristics	CG ( n=25 )	Patients ( n=34 )	p-value
THA	Age (years), Mean±SD	69.28±10.11	67.62±8.28	0.540
	Gender, male/female n (%)	13(52%)/12(48%)	15(44%)/19(56%)	0.605
	Weight (kg), Mean±SD	79.40±13.08	82.32±17.73	0.371
	Height (mm), Mean±SD	165.04±9.46	165.79±8.80	0.754
	ΒΜI (kg/mm^2^), Mean±SD	29.00±3.00	29.72±4.31	0.482
	Operated leg (right/left) n (%)	---	20(58.8%)/14(41.2%)	---

The parameters of the spine and the pelvis in the patients surveyed preoperatively and at four months after THA are summarized in Tables 2-5. At four months postoperatively, patients presented significantly decreased values compared with the preoperative values, but values improved to normal for the lordotic angle (°; 46.45→44.19, p = 0.006), trunk torsion (°; 8.28→5.03, p = 0.031), pelvic inclination (°; 22.31→19.07, p = 0.030), pelvic obliquity (°; 3.97→2.52, p = 0.018), pelvic obliquity (mm; 5.59→3.77, p = 0.013), and pelvis rotation (°; 3.69→0.72, p = 0.001).

**Table 2:** Comparison of parameters of the spine for patients with THA between preoperative and postoperative four-month values THA: total hip arthroplasty, MFU: months follow-up, SD: standard deviation, rms: root mean square, pre-oper: preoperative, post-oper: postoperative

THA PRE-OPERATION vs POST-OPERATION (4MFU)	Mean	SD
fleche cervicale (mm) pre-oper	92.61	28.35
fleche cervicale (mm) post-oper 4	93.63	27.33
fleche lombaire (mm) pre-oper	28.30	19.38
fleche lombaire (mm) post-oper 4	30.54	18.26
kyphotic angle (°) pre-oper	58.30	11.64
kyphotic angle (°) post-oper 4	60.83	9.75
lordotic angle (°) pre-oper	46.45	11.57
lordotic angle (°) post-oper 4	44.19	11.13
sagittal imbalance (°) pre-oper	8.55	5.47
sagittal imbalance (°) post-oper 4	7.52	5.04
sagittal imbalance (mm) pre-oper	65.12	43.41
sagittal imbalance (mm) post-oper 4	57.11	40.55
coronal imbalance (°) pre-oper	1.10	0.86
coronal imbalance (°) post-oper 4	1.62	1.35
coronal imbalance (mm) pre-oper	7.99	6.01
coronal imbalance (mm) post-oper 4	12.43	10.08
apical deviation rms (mm) pre-oper	5.99	3.83
apical deviation rms (mm) post-oper 4	5.31	2.82
apical deviation amplitude (mm) pre-oper	13.41	5.85
apical deviation amplitude (mm) post-oper 4	11.90	4.86
apical deviation max (mm) pre-oper	10.66	5.70
apical deviation max (mm) post-oper 4	9.59	4.48
scoliosis angle (°) pre-oper	16.34	7.11
scoliosis angle (°) post-oper 4	15.28	6.85
vertebral rotation rms (°) pre-oper	5.14	2.57
vertebral rotation rms (°) post-oper 4	4.88	2.36
vertebral rotation amplitude (°) pre-oper	11.45	4.35
vertebral rotation amplitude (°) post-oper 4	11.28	5.14
vertebral rotation max (°) pre-oper	9.41	3.49
vertebral rotation max (°) post-oper 4	9.10	3.85
trunk torsion (°) pre-oper	8.28	7.66
trunk torsion (°) post-oper 4	5.03	4.67

**Table 3:** Comparison of parameters of the spine for patients with THA between preoperative and postoperative four-month values THA: total hip arthroplasty, MFU: months follow-up, SD: standard deviation, rms: root mean square, CI: confidence interval

THA PRE-OPERATION vs POST-OPERATION (4MFU)	Mean	SD	95% CI		p-value
			Lower	Upper	
fleche cervicale (mm)0 - fleche cervicale (mm)4	-1.03	23.80	-10.08	8.03	0.818
fleche lombaire (mm)0 - fleche lombaire (mm)4	-2.25	11.44	-6.60	2.11	0.299
kyphotic angle (°)0 - kyphotic angle (°)4	-2.53	8.86	-5.90	0.84	0.135
lordotic angle (°)0 - lordotic angle (°)4	2.26	4.08	0.71	3.81	0.006
sagittal imbalance (°)0 - sagittal imbalance (°)4	1.03	3.84	-0.43	2.49	0.161
sagittal imbalance (mm)0 - sagittal imbalance (mm)4	8.01	29.16	-3.09	19.10	0.150
coronal imbalance (°)0 - coronal imbalance (°)4	-0.52	1.50	-1.09	0.05	0.074
coronal imbalance (mm)0 - coronal imbalance (mm)4	-4.44	11.81	-8.93	0.05	0.053
apical deviation rms (mm)0 - apical deviation rms (mm)4	0.68	3.67	-0.72	2.08	0.328
apical deviation amplitude (mm)0 - apical deviation amplitude (mm)4	1.52	5.49	-0.57	3.61	0.148
apical deviation max (mm)0 - apical deviation max (mm)4	1.07	5.46	-1.01	3.14	0.301
scoliosis angle (°)0 - scoliosis angle (°)4	1.07	5.96	-1.20	3.34	0.342
vertebral rotation rms (°)0 - vertebral rotation rms (°)4	0.27	2.87	-0.83	1.36	0.622
vertebral rotation amplitude (°)0 - vertebral rotation amplitude (°)4	0.17	4.38	-1.49	1.84	0.834
vertebral rotation max (°)0 - vertebral rotation max (°)4	0.31	4.23	-1.30	1.92	0.696
trunk torsion (°)0 - trunk torsion (°)4	3.24	7.70	0.31	6.17	0.031

**Table 4:** Comparison of parameters of the pelvis for patients with THA between preoperative and postoperative four-month values THA: total hip arthroplasty, MFU: months follow-up, SD: standard deviation

THA PRE-OPERATION vs POST-OPERATION (4MFU)	Mean	SD
pelvic inclination symmetry line (°) pre-oper	22.31	10.84
pelvic inclination symmetry line (°) post-oper 4	19.07	10.76
pelvic inclination dimples (°) pre-oper	18.86	8.23
pelvic inclination dimples (°) post-oper 4	17.38	8.42
pelvic torsion (°) pre-oper	3.14	1.73
pelvic torsion (°) post-oper 4	2.79	1.66
pelvic obliquity (°) pre-oper	3.97	3.42
pelvic obliquity (°) post-oper 4	2.52	2.18
pelvic obliquity (mm) pre-oper	5.59	4.20
pelvic obliquity (mm) post-oper 4	3.77	3.27
pelvis rotation (°) pre-oper	3.69	4.02
pelvis rotation (°) post-oper 4	0.72	1.56

**Table 5:** Comparison of parameters of the pelvis for patients with THA between preoperative and postoperative four-month values THA: total hip arthroplasty, MFU: months follow-up, SD: standard deviation, CI: confidence interval

THA PRE-OPERATION vs POST-OPERATION (4MFU)	Mean	SD	95% CI		p-value
			Lower	Upper	
pelvic inclination symmetry line (°)0 - pelvic inclination symmetry line (°)4	3.24	7.62	0.34	6.14	0.030
pelvic inclination dimples (°)0 - pelvic inclination dimples (°)4	1.48	4.75	-0.32	3.29	0.104
pelvic torsion (°)0 - pelvic torsion (°)4	0.34	1.95	-0.40	1.09	0.349
pelvic obliquity (°)0 - pelvic obliquity (°)4	1.45	3.11	0.26	2.63	0.018
pelvic obliquity (mm)0 - pelvic obliquity (mm)4	1.82	3.67	0.42	3.21	0.013
pelvis rotation (°)0 - pelvis rotation (°)4	2.97	4.08	1.42	4.52	0.001

The parameters of the spine and the pelvis in the patients surveyed both preoperatively and at 12 months after THA are summarized in Tables 6-9. At 12 months postoperatively, patients presented significantly decreased values compared with the preoperative values, and values improved to normal only for pelvic obliquity (°; 4.05→2.35, p = 0.010) and pelvic obliquity (mm; 6.03→3.61, p = 0.009).

**Table 6:** Comparison of parameters of the spine for patients with THA between preoperative and postoperative 12-month values THA: total hip arthroplasty, MFU: months follow-up, SD: standard deviation, rms: root mean square, pre-oper: preoperative, post-oper: postoperative

THA PRE-OPERATION vs POST-OPERATION (12MFU)	Mean	SD
fleche cervicale (mm) pre-oper	85.38	30.39
fleche cervicale (mm) post-oper 12	91.57	31.45
fleche lombaire (mm) pre-oper	23.54	22.14
fleche lombaire (mm) post-oper 12	28.23	24.63
kyphotic angle (°) pre-oper	57.12	14.33
kyphotic angle (°) post-oper 12	61.43	11.17
lordotic angle (°) pre-oper	47.98	11.44
lordotic angle (°) post-oper 12	46.75	11.38
sagittal imbalance (°) pre-oper	8.97	5.33
sagittal imbalance (°) post-oper 12	8.03	6.08
sagittal imbalance (mm) pre-oper	69.07	42.09
sagittal imbalance (mm) post-oper 12	62.58	46.81
coronal imbalance (°) pre-oper	1.55	1.05
coronal imbalance (°) post-oper 12	1.50	1.19
coronal imbalance (mm) pre-oper	11.44	7.53
coronal imbalance (mm) post-oper 12	10.98	8.18
apical deviation rms (mm) pre-oper	6.85	4.09
apical deviation rms (mm) post-oper 12	6.76	2.78
apical deviation amplitude (mm) pre-oper	14.50	6.19
apical deviation amplitude (mm) post-oper 12	15.80	7.18
apical deviation max (mm) pre-oper	11.75	6.21
apical deviation max (mm) post-oper 12	12.35	4.98
scoliosis angle (°) pre-oper	16.95	7.98
scoliosis angle (°) post-oper 12	17.10	7.26
vertebral rotation rms (°) pre-oper	5.35	2.86
vertebral rotation rms (°) post-oper 12	4.79	2.23
vertebral rotation amplitude (°) pre-oper	12.15	4.77
vertebral rotation amplitude (°) post-oper 12	12.45	4.27
vertebral rotation max (°) pre-oper	9.60	3.59
vertebral rotation max (°) post-oper 12	9.40	3.33
trunk torsion (°) pre-oper	7.80	6.89
trunk torsion (°) post-oper 12	6.55	4.95

**Table 7:** Comparison of parameters of the spine for patients with THA between preoperative and postoperative 12-month values THA: total hip arthroplasty, MFU: months follow-up, SD: standard deviation, rms: root mean square, CI: confidence interval

THA PRE-OPERATION vs POST-OPERATION (12MFU)	Mean	SD	95% CI		p-value
			Lower	Upper	
fleche cervicale (mm)0 - fleche cervicale (mm)12	-6.19	24.90	-17.84	50.46	0.280
fleche lombaire (mm)0 - fleche lombaire (mm)12	-4.70	13.63	-11.07	10.68	0.140
kyphotic angle (°)0 - kyphotic angle (°)12	-4.31	10.43	-9.19	0.57	0.080
lordotic angle (°)0 - lordotic angle (°)12	1.24	3.37	-0.34	20.81	0.117
sagittal imbalance (°)0 - sagittal imbalance (°)12	0.94	4.72	-1.27	30.15	0.384
sagittal imbalance (mm)0 - sagittal imbalance (mm)12	6.49	34.12	-9.48	220.46	0.406
coronal imbalance (°)0 - coronal imbalance (°)12	0.05	1.57	-0.69	0.79	0.888
coronal imbalance (mm)0 - coronal imbalance (mm)12	0.46	12.15	-5.22	6.14	0.867
apical deviation rms (mm)0 - apical deviation rms (mm)12	0.09	4.11	-1.84	2.01	0.927
apical deviation amplitude (mm)0 - apical deviation amplitude (mm)12	-1.30	6.94	-4.55	1.95	0.413
apical deviation max (mm)0 - apical deviation max (mm)12	-0.60	6.24	-3.52	2.32	0.672
scoliosis angle (°)0 - scoliosis angle (°)12	-0.15	5.92	-2.92	2.62	0.911
vertebral rotation rms (°)0 - vertebral rotation rms (°)12	0.56	3.01	-0.85	1.96	0.419
vertebral rotation amplitude (°)0 - vertebral rotation amplitude (°)12	-0.30	3.85	-2.10	1.50	0.732
vertebral rotation max (°)0 - vertebral rotation max (°)12	0.20	3.69	-1.53	1.93	0.811
trunk torsion (°)0 - trunk torsion (°)12	1.25	6.33	-1.71	4.21	0.388

**Table 8:** Comparison of parameters of the pelvis for patients with THA between preoperative and postoperative 12-month values THA: total hip arthroplasty, MFU: months follow-up, SD: standard deviation, pre-oper: preoperative, post-oper: postoperative

THA PRE-OPERATION vs POST-OPERATION (12MFU)	Mean	SD
pelvic inclination symmetry line (°) pre-oper	24.90	10.79
pelvic inclination symmetry line (°) post-oper 12	23.50	10.32
pelvic inclination dimples (°) pre-oper	21.05	6.57
pelvic inclination dimples (°) post-oper 12	20.65	7.26
pelvic torsion (°) pre-oper	3.55	1.67
pelvic torsion (°) post-oper 12	3.15	2.13
pelvic obliquity (°) pre-oper	4.05	3.35
pelvic obliquity (°) post-oper 12	2.35	3.48
pelvic obliquity (mm) pre-oper	6.03	5.12
pelvic obliquity (mm) post-oper 12	3.61	4.96
pelvis rotation (°) pre-oper	3.50	4.15
pelvis rotation (°) post-oper 12	1.50	1.73

**Table 9:** Comparison of parameters of the pelvis for patients with THA between preoperative and postoperative 12-month values THA: total hip arthroplasty, MFU: months follow-up, SD: standard deviation, CI: confidence interval

THA PRE-OPERATION vs POST-OPERATION (12MFU)	Mean	SD	95% CI		p-value
			Lower	Upper	
pelvic inclination symmetry line (°)0 - pelvic inclination symmetry line (°)12	1.40	5.67	-1.25	4.05	0.283
pelvic inclination dimples (°)0 - pelvic inclination dimples (°)12	0.40	4.81	-1.85	2.65	0.714
pelvic torsion (°)0 - pelvic torsion (°)12	0.40	2.28	-0.67	1.47	0.442
pelvic obliquity (°)0 - pelvic obliquity (°)12	1.70	2.64	0.47	2.93	0.010
pelvic obliquity (mm)0 - pelvic obliquity (mm)12	2.42	3.74	0.67	4.17	0.009
pelvis rotation (°)0 - pelvis rotation (°)12	2.00	5.01	-0.34	4.34	0.090

Patients with TKA

Another group of 45 patients with 34 women, 11 men, and a mean age of 72.42 ± 7.00 years (range, 54-90 years) was surveyed preoperatively. Of these patients, 24 were surveyed at four and 12 months postoperatively, 12 patients were surveyed only at four months postoperatively, and nine patients were surveyed only at 12 months postoperatively. There were 20 patients who underwent an operation for the right knee and 25 for the left knee. Four orthopedic surgeons carried out TKA. Other characteristics of these patients are a mean weight of 79.87 ± 13.79 kg, a mean height of 162.16 ± 5.89 mm, and a mean BMI of 30.36 ± 4.49 kg/m^2^. Table 10 summarizes the homogeneity of the demographic characteristics between the CG and the patients with TKA. No statistically significant differences were noted between the CG and patients with TKA except for gender.

**Table 10:** Homogeneity of demographic characteristics between the CG and patients with TKA CG: control group, TKA: total knee arthroplasty, SD: standard deviation, BMI: body mass index

	Characteristics	CG ( n=25 )	Patients ( n=45 )	p-value
TKA	Age (years), Mean±SD	69.28±10.11	72.42±7.00	0.175
	Gender, male/female n (%)	13(52%)/12(48%)	11(24.4%)/34(75.6%)	0.034
	Weight (kg), Mean±SD	79.40±13.08	79.87±13.79	0.891
	Height (mm), Mean±SD	165.04±9.46	162.16±5.89	0.176
	ΒΜΙ (kg/m^2^), Mean±SD	29.00±3.00	30.36±4.49	0.136
	Operated leg (right/left) n (%)	---	20(44.4%)/25(55.6%)	---

The parameters of the spine and the pelvis in the patients surveyed both preoperatively and four months after TKA are summarized in Tables 11-14. At four months postoperatively, patients presented with significantly decreased values compared with the preoperative values, but values improved to normal for the lordotic angle (°; 49.69→46.78, p = 0.002), pelvic inclination (°; 26.50→24.33, p = 0.032), pelvic obliquity (°; 2.78→1.86, p = 0.008) and pelvic obliquity (mm; 4.02→2.47, p = 0.005). However, four months postoperatively, patients presented significantly increased values compared with the preoperative values and worse values than normal values for the fleche cervicale (mm; 82.71→94.09, p = 0.004) and vertebral rotation (°; 4.81→5.66, p = 0.046).

**Table 11:** Comparison of parameters of the spine for patients with TKA between preoperative and postoperative four-month values TKA: total knee arthroplasty, MFU: months follow-up, SD: standard deviation, rms: root mean square, pre-oper: preoperative, post-oper: postoperative

TΚA PRE-OPERATION vs POST-OPERATION (4MFU)	Mean	SD
fleche cervicale (mm) pre-oper	82.71	25.52
fleche cervicale (mm) post-oper 4	94.09	28.01
fleche lombaire (mm) pre-oper	31.62	19.24
fleche lombaire (mm) post-oper 4	31.15	17.70
kyphotic angle (°) pre-oper	60.80	14.03
kyphotic angle (°) post-oper 4	63.89	14.04
lordotic angle (°) pre-oper	49.69	17.41
lordotic angle (°) post-oper 4	46.78	15.57
sagittal imbalance (°) pre-oper	7.28	5.07
sagittal imbalance (°) post-oper 4	7.74	4.04
sagittal imbalance (mm) pre-oper	53.24	37.61
sagittal imbalance (mm) post-oper 4	57.59	31.97
coronal imbalance (°) pre-oper	1.08	1.05
coronal imbalance (°) post-oper 4	1.06	1.04
coronal imbalance (mm) pre-oper	7.87	7.13
coronal imbalance (mm) post-oper 4	7.99	6.94
apical deviation rms (mm) pre-oper	6.69	3.67
apical deviation rms (mm) post-oper 4	6.48	3.28
apical deviation amplitude (mm) pre-oper	13.11	5.38
apical deviation amplitude (mm) post-oper 4	13.17	5.13
apical deviation max (mm) pre-oper	11.19	5.38
apical deviation max (mm) post-oper 4	10.50	5.29
scoliosis angle (°) pre-oper	16.58	5.92
scoliosis angle (°) post-oper 4	16.83	6.20
vertebral rotation rms (°) pre-oper	4.81	2.73
vertebral rotation rms (°) post-oper 4	5.66	3.51
vertebral rotation amplitude (°) pre-oper	11.67	5.34
vertebral rotation amplitude (°) post-oper 4	11.94	4.77
vertebral rotation max (°) pre-oper	8.94	4.36
vertebral rotation max (°) post-oper 4	10.28	4.68
trunk torsion (°) pre-oper	7.33	6.54
trunk torsion (°) post-oper 4	7.75	4.78

**Table 12:** Comparison of parameters of the spine for patients with TKA between preoperative and postoperative four-month values TKA: total knee arthroplasty, MFU: months follow-up, SD: standard deviation, rms: root mean square, CI: confidence interval

TΚA PRE-OPERATION vs POST-OPERATION (4MFU)	Mean	SD	95% CI		p-value
			Lower	Upper	
fleche cervicale (mm)0 - fleche cervicale (mm)4	-11.39	22.11	-18.87	-3.91	0.004
fleche lombaire (mm)0 - fleche lombaire (mm)4	0.47	6.42	-1.70	2.64	0.665
kyphotic angle (°)0 - kyphotic angle (°)4	-3.08	10.11	-6.50	0.34	0.076
lordotic angle (°)0 - lordotic angle (°)4	2.91	5.08	1.20	4.63	0.002
sagittal imbalance (°)0 - sagittal imbalance (°)4	-0.46	2.91	-1.45	0.52	0.348
sagittal imbalance (mm)0 - sagittal imbalance (mm)4	-4.35	22.17	-11.85	3.15	0.247
coronal imbalance (°)0 - coronal imbalance (°)4	0.03	1.21	-0.38	0.44	0.891
coronal imbalance (mm)0 - coronal imbalance (mm)4	-0.12	8.03	-2.84	2.60	0.928
apical deviation rms (mm)0 - apical deviation rms (mm)4	0.22	2.87	-0.75	1.19	0.653
apical deviation amplitude (mm)0 - apical deviation amplitude (mm)4	-0.06	4.59	-1.61	1.50	0.942
apical deviation max (mm)0 - apical deviation max (mm)4	0.69	4.96	-0.98	2.37	0.406
scoliosis angle (°)0 - scoliosis angle (°)4	-0.25	4.70	-1.84	1.34	0.752
vertebral rotation rms (°)0 - vertebral rotation rms (°)4	-0.85	2.46	-1.68	-0.02	0.046
vertebral rotation amplitude (°)0 - vertebral rotation amplitude (°)4	-0.28	3.90	-1.60	1.04	0.672
vertebral rotation max (°)0 - vertebral rotation max (°)4	-1.33	3.97	-2.68	0.01	0.052
trunk torsion (°)0 - trunk torsion (°)4	-0.42	6.84	-2.73	1.90	0.717

**Table 13:** Comparison of parameters of the pelvis for patients with TKA between preoperative and postoperative four-month values TKA: total knee arthroplasty, MFU: months follow-up, SD: standard deviation, pre-oper: preoperative, post-oper: postoperative

TΚA PRE-OPERATION vs POST-OPERATION (4MFU)	Mean	SD
pelvic inclination symmetry line (°) pre-oper	26.50	17.73
pelvic inclination symmetry line (°) post-oper 4	24.33	17.36
pelvic inclination dimples (°) pre-oper	20.83	13.00
pelvic inclination dimples (°) post-oper 4	19.44	15.07
pelvic torsion (°) pre-oper	2.89	2.35
pelvic torsion (°) post-oper 4	2.72	1.72
pelvic obliquity (°) pre-oper	2.78	2.59
pelvic obliquity (°) post-oper 4	1.86	2.26
pelvic obliquity (mm) pre-oper	4.02	3.88
pelvic obliquity (mm) post-oper 4	2.47	2.77
pelvis rotation (°) pre-oper	3.31	4.47
pelvis rotation (°) post-oper 4	1.97	2.67

**Table 14:** Comparison of parameters of the pelvis for patients with TKA between preoperative and postoperative four-month values TKA: total knee arthroplasty, MFU: months follow-up, SD: standard deviation, CI: confidence interval

TΚA PRE-OPERATION vs POST-OPERATION (4MFU)	Mean	SD	95% CI		p-value
			Lower	Upper	
pelvic inclination symmetry line (°)0 - pelvic inclination symmetry line (°)4	2.17	5.81	0.20	4.13	0.032
pelvic inclination dimples (°)0 - pelvic inclination dimples (°)4	1.39	6.59	-0.84	3.62	0.214
pelvic torsion (°)0 - pelvic torsion (°)4	0.17	2.24	-0.59	0.92	0.657
pelvic obliquity (°)0 - pelvic obliquity (°)4	0.92	1.95	0.26	1.58	0.008
pelvic obliquity (mm)0 - pelvic obliquity (mm)4	1.55	3.08	0.51	2.59	0.005
pelvis rotation (°)0 - pelvis rotation (°)4	1.33	4.48	-0.18	2.85	0.083

The parameters of the spine and the pelvis in the patients surveyed both preoperatively and at 12 months after TKA are summarized in Tables 15-18. At 12 months postoperatively, patients presented significantly decreased values compared with the preoperative values, and values improved to normal only for pelvic obliquity (°; 3.58→1.94, p < 0.001) and pelvic obliquity (mm; 4.93→2.59, p = 0.001).

**Table 15:** Comparison of parameters of the pelvis for patients with TKA between preoperative and postoperative 12-month values TKA: total knee arthroplasty, MFU: months follow-up, SD: standard deviation, rms: root mean square, pre-oper: preoperative, post-oper: postoperative

TΚA PRE-OPERATION vs POST-OPERATION (12MFU)	Mean	SD
fleche cervicale (mm) pre-oper	85.10	24.38
fleche cervicale (mm) post-oper 12	89.61	30.00
fleche lombaire (mm) pre-oper	31.80	16.12
fleche lombaire (mm) post-oper 12	30.15	16.17
kyphotic angle (°) pre-oper	62.33	12.37
kyphotic angle (°) post-oper 12	63.60	12.00
lordotic angle (°) pre-oper	46.94	15.09
lordotic angle (°) post-oper 12	46.48	15.05
sagittal imbalance (°) pre-oper	7.14	4.36
sagittal imbalance (°) post-oper 12	7.84	4.86
sagittal imbalance (mm) pre-oper	52.18	32.03
sagittal imbalance (mm) post-oper 12	58.01	36.52
coronal imbalance (°) pre-oper	1.39	1.12
coronal imbalance (°) post-oper 12	1.42	1.20
coronal imbalance (mm) pre-oper	9.91	7.37
coronal imbalance (mm) post-oper 12	10.44	8.48
apical deviation rms (mm) pre-oper	6.86	3.72
apical deviation rms (mm) post-oper 12	5.78	2.82
apical deviation amplitude (mm) pre-oper	14.30	6.34
apical deviation amplitude (mm) post-oper 12	12.88	4.72
apical deviation max (mm) pre-oper	12.42	6.29
apical deviation max (mm) post-oper 12	10.70	4.83
scoliosis angle (°) pre-oper	18.09	6.41
scoliosis angle (°) post-oper 12	16.30	5.77
vertebral rotation rms (°) pre-oper	4.88	2.46
vertebral rotation rms (°) post-oper 12	5.49	2.75
vertebral rotation amplitude (°) pre-oper	12.36	5.06
vertebral rotation amplitude (°) post-oper 12	11.64	4.36
vertebral rotation max (°) pre-oper	9.30	3.93
vertebral rotation max (°)post-oper 12	9.76	4.19
trunk torsion (°) pre-oper	7.09	5.89
trunk torsion (°) post-oper 12	7.73	6.10

**Table 16:** Comparison of parameters of the pelvis for patients with TKA between preoperative and postoperative 12-month values TKA: total knee arthroplasty, MFU: months follow-up, SD: standard deviation, rms: root mean square, CI: confidence interval

TΚA PRE-OPERATION vs POST-OPERATION (12MFU)	Mean	SD	95% CI		p-value
			Lower	Upper	
fleche cervicale (mm)0 - fleche cervicale (mm)12	-4.51	22.84	-12.61	3.59	0.265
fleche lombaire (mm)0 - fleche lombaire (mm)12	1.65	8.38	-1.32	4.62	0.267
kyphotic angle (°)0 - kyphotic angle (°)12	-1.27	8.97	-4.45	1.91	0.423
lordotic angle (°)0 - lordotic angle (°)12	0.47	4.85	-1.25	2.19	0.584
sagittal imbalance (°)0 - sagittal imbalance (°)12	-0.70	3.22	-1.84	0.44	0.221
sagittal imbalance (mm)0 - sagittal imbalance (mm)12	-5.83	25.92	-15.02	3.36	0.206
coronal imbalance (°)0 - coronal imbalance (°)12	-0.03	1.55	-0.58	0.52	0.911
coronal imbalance (mm)0 - coronal imbalance (mm)12	-0.53	10.25	-4.17	3.10	0.767
apical deviation rms (mm)0 - apical deviation rms (mm)12	1.09	3.35	-0.10	2.27	0.091
apical deviation amplitude (mm)0 - apical deviation amplitude (mm)12	1.42	4.96	-0.34	3.18	0.109
apical deviation max (mm)0 - apical deviation max (mm)12	1.73	5.66	-0.28	3.73	0.089
scoliosis angle (°)0 - scoliosis angle (°)12	1.79	5.42	-0.13	3.71	0.067
vertebral rotation rms (°)0 - vertebral rotation rms (°)12	-0.61	3.10	-1.71	0.49	0.265
vertebral rotation amplitude (°)0 - vertebral rotation amplitude (°)12	0.73	5.92	-1.37	2.83	0.485
vertebral rotation max (°)0 - vertebral rotation max (°)12	-0.45	4.81	-2.16	1.25	0.591
trunk torsion (°)0 - trunk torsion (°)12	-0.64	4.88	-2.37	1.09	0.459

**Table 17:** Comparison of parameters of the pelvis for patients with TKA between preoperative and postoperative 12-month values TKA: total knee arthroplasty, MFU: months follow-up, SD: standard deviation, pre-oper: preoperative, post-oper: postoperative

TΚA PRE-OPERATION vs POST-OPERATION (12MFU)	Mean	SD
pelvic inclination symmetry line (°) pre-oper	21.18	15.63
pelvic inclination symmetry line (°) post-oper 12	21.09	15.35
pelvic inclination dimples (°) pre-oper	17.64	11.79
pelvic inclination dimples (°) post-oper 12	17.27	12.17
pelvic torsion (°) pre-oper	2.42	2.03
pelvic torsion (°) post-oper 12	2.33	1.51
pelvic obliquity (°) pre-oper	3.58	2.94
pelvic obliquity (°) post-oper 12	1.94	2.08
pelvic obliquity (mm) pre-oper	4.93	4.03
pelvic obliquity (mm) post-oper 12	2.59	2.87
pelvis rotation (°) pre-oper	3.18	4.45
pelvis rotation (°) post-oper 12	2.27	3.20

**Table 18:** Comparison of parameters of the pelvis for patients with TKA between preoperative and postoperative 12-month values TKA: total knee arthroplasty, MFU: months follow-up, SD: standard deviation, CI: confidence interval

TΚA PRE-OPERATION vs POST-OPERATION (12MFU)	Mean	SD	95% CI		p-value
			Lower	Upper	
pelvic inclination symmetry line (°)0 - pelvic inclination symmetry line (°)12	0.09	5.33	-1.80	1.98	0.923
pelvic inclination dimples (°)0 - pelvic inclination dimples (°)12	0.36	3.83	-0.99	1.72	0.589
pelvic torsion (°)0 - pelvic torsion (°)12	0.09	2.20	-0.69	0.87	0.814
pelvic obliquity (°)0 - pelvic obliquity (°)12	1.64	2.37	0.80	2.48	<0.001
pelvic obliquity (mm)0 - pelvic obliquity (mm)12	2.34	3.49	1.10	3.58	0.001
pelvis rotation (°)0 - pelvis rotation (°)12	0.91	5.06	-0.89	2.70	0.310

## Discussion

We think that the relationships between the hip and knee OA and the disordered morphology of the spine and pelvis, as well as their possible causal relationship, are very interesting topics in orthopedics. We have tried to address this issue in two steps. The first step was to study the trunk morphology in two groups of patients with hip and knee OA and to compare them with a CG. The results of this study showed that the spine and pelvis morphology is actually disturbed in patients with hip and knee OA [1]. The same results were also mentioned in other studies [2-7]. The second step was to study the same groups of patients with hip or knee OA undergoing THA or TKA and to study the effect of these operations on the disturbed morphology of the spine and the pelvis.

Compared to the CG, the patients with hip OA had a greater forward inclination of the spine, increased scoliosis, more vertebral rotation and trunk torsion, and greater obliquity of the pelvis in the frontal plane [1]. The results of this study at four months after THA revealed that the lordotic angle, trunk torsion, pelvic inclination, pelvic obliquity, and pelvis rotation were improved to normal levels. In the long term, at 12 months after THA, only the pelvic obliquity was improved to normal levels.

Compared to the CG, the patients with knee OA had a greater forward inclination of the spine, increased scoliosis, apical deviation, more vertebral rotation and trunk torsion, and greater obliquity of the pelvis in the frontal plane [1]. The results of this study at four months after TKA revealed that the lordotic angle, pelvic inclination, and pelvic obliquity were improved to normal levels. However, the fleche cervicale and the vertebral rotation were worse. In the long term at 12 months after TKA, only the pelvic obliquity was improved to normal levels.

After THA there was a temporary improvement of the morphological parameters of the spine and pelvis at four months postoperatively. However, at 12 months after THA, these positive effects were eliminated, and the preoperative pathological morphology of the spine and pelvis returned. The only exception was the improved pelvic obliquity at the frontal level, which remained at 12 months.

In the case of TKA, it did not have an overall positive effect on the parameters of the spine. In the pelvis, despite the initial improvement of the sagittal inclination at four months, it was eventually eliminated at 12 months. Again, the only exception was the improved pelvic obliquity at the frontal level, which remained at 12 months. The improved pelvic obliquity at 12 months postoperatively in both groups of patients could be attributed to the fact that this parameter is largely determined by leg-length inequality. Therefore, the correction of this inequality after THA and TKA was enough to correct the pelvic obliquity at 12 months.

Thus, although THA and TKA operations repaired the hip and knee OA, they could not repair the disturbed morphology of the spine and pelvis in the long run. This means that the hip and knee OA could not be responsible for the disturbed morphology of the spine and the pelvis, but other causes and mechanisms should be responsible for this morphology. These mechanisms apparently persisted postoperatively, resulting in the recurrence of the same problems of trunk morphology at 12 months. A proposed etiological mechanism could be the asymmetric action of the trunk muscles in these patients, which pre-exist and persist postoperatively, resulting in the recurrence of disturbed morphology of the spine and pelvis in patients undergoing THA and TKA. In fact, a similar pathogenetic mechanism has been described in patients with scoliosis [10-12].

From the results of this study, it cannot be ruled out that the disturbed morphology of the spine and pelvis could be responsible for the appearance of hip and knee OA, but further studies are needed to determine if it is truly the cause. There may also be other factors than disordered trunk morphology that continue to exist after THA or TKA and could be responsible for the appearance of hip and knee OA.

Limitations

There were some limitations to this study. The first was that the number of patients was limited. The second was that not all the patients were examined at four and 12 months. The third was that the estimations using the Diers Formetric four-D system were done by only one examiner.

## Conclusions

An interesting and unanswered issue in orthopedics is the relationship between the hip and knee OA and the occurrence of disturbed morphology of the trunk, as well as their possible etiological relationship. From this study, it appears that hip and knee OA is not responsible for the disturbed morphology of the trunk. On the other hand, it is still unclear whether the disturbed trunk morphology is responsible for causing the hip and knee OA. Other studies are necessary to provide an answer to this interesting question.
